# Acoustic recordings provide detailed information regarding the behavior of cryptic wildlife to support conservation translocations

**DOI:** 10.1038/s41598-019-41455-z

**Published:** 2019-03-26

**Authors:** Xiao Yan, Hemin Zhang, Desheng Li, Daifu Wu, Shiqiang Zhou, Mengmeng Sun, Haiping Hu, Xiaoqiang Liu, Shijie Mou, Shengshan He, Megan A. Owen, Yan Huang

**Affiliations:** 1 China Conservation and Research Centre for the Giant Panda, Wolong, Sichuan 623006 China; 2 Institute for Conservation Research, San Diego Zoo Global, California, United States; 3 Key Laboratory of Biodiversity Science and Ecological Engineering of Ministry of Education, School of Life Sciences, Beijing Normal University, Beijing, China

## Abstract

For translocated animals, behavioral competence may be key to post-release survival. However, monitoring behavior is typically limited to tracking movements or inferring behavior at a gross scale via collar-mounted sensors. Animal-bourne acoustic monitoring may provide a unique opportunity to monitor behavior at a finer scale. The giant panda is an elusive species of Ursid that is *vulnerable* to extinction. Translocation is an important aspect of the species’ recovery, and survival and recruitment for pandas likely hinge on behavioral competence. Here we tested the efficacy of a collar-mounted acoustic recording unit (ARU) to remotely monitor the behavior of panda mothers and their dependent young. We found that trained human listeners could reliably identify 10 behaviors from acoustic recordings. Through visual inspection of spectrograms we further identified 5 behavioral categories that may be detectable by automated pattern recognition, an approach that is essential for the practical application of ARU. These results suggest that ARU are a viable method for remotely observing behaviors, including feeding. With targeted effort directed towards instrumentation and computing advances, ARU could be used to document how behavioral competence supports or challenges post-release survival and recruitment, and allow for research findings to be adaptively integrated into future translocation efforts.

## Introduction

For translocated animals, behavioral competence (i.e., the capacity for an individual to engage in behaviors that support reproduction and survival in a range of contexts) can be fundamentally important to post-release survival and recruitment^1^. However, direct observation of wide-ranging and/or cryptic species is extremely difficult, and monitoring behavior post-release is typically limited to tracking movements^2^ or inferring behavior at a gross scale through data collected via collar-mounted accelerometers or environmental sensors^3^. This inability to monitor behavior at a finer scale impedes identification of the mechanisms responsible for translocation success and failure, including post-release effects^4^, and thus limits the adaptive development of strategies designed to improve the effectiveness of pre-release training and post-release outcomes^5^. For example, if inadequate anti-predator behavior is responsible for disproportionately high rates of mortality among translocated individuals, pre-release training may target appropriate anti-predator behavioral skills^6,7^. The same approach could be taken in cases where social incompetence results in recruitment failure or when inadequate competitive behavior results in serious injury during competition for mates^8^. Additionally, understanding the behavioral repertoire of conspecific mentors during the pre-release training period can ensure that release candidates are exposed to appropriate exemplar behavior. Because direct observation by humans may compromise natural behaviors of release candidates, remote observation is often required. In tandem with the identification of other factors that may contribute to translocation failures (e.g., release timing, age at release, and release site characteristics)^9^, knowledge of behavioral inadequacy can inform future pre-release training and translocation efforts.

Over the past decade, technological advancements in bio-logging and environmental platforms, data management and computation, have greatly facilitated the practical application of animal-bourne and environmental sensors^10,11^. In the realm of acoustics sensors, these advancements have made the application of acoustic monitoring to ecology and conservation far more feasible and valuable^12,13^, with impressive initiatives and results from studies focused on marine species^14^ and in the realm of soundscape ecology; a developing field based on the information content and dynamics of sound in natural systems^15^. Soundscape ecology is often focused on the use of passive acoustic monitoring (PAM) to characterize the biotic and abiotic acoustic dynamics of particular habitats and to quantify the animals within them^16,17^. PAM is generally habitat-focused in that it involves installing an acoustic recording array in an environment, and tracking biodiversity, phenology or other characteristics over time, in that locale^18,19^. As such, habitat-anchored PAM has limited utility for monitoring individual animals through space and over time; a critical aspect of post-release monitoring. However, focused development of bio-logging technologies, including sensors, supporting data acquisition platforms, sensor housings and data processing software^10^ will facilitate the development of animal-bourne approaches to acoustic monitoring in marine^20^ and terrestrial environments^21^.

Animal-bourne acoustic monitoring fundamentally redirects the focus of data from the environment to an individual animal and the sounds that it makes and experiences as it moves through space and over time^20,21^. By moving the recording platform to the animal, opportunities emerge for capturing a wider range of behaviors, and these can be important indicators of normal life function or challenges to survival^21^, including interactions with conspecifics, heterospecifics and physiological indications of stress^22^. While the communicative function of a wide-range of acoustic signals and cues has been explored extensively through behavioral and ecological research^23^, the information content of incidental sound–a common byproduct of many aspects of an animal’s life—has been generally understudied^24^. Incidental sound, which may include byproducts of non-communicative behaviors (e.g., foraging^25^ and resting), physical processes (e.g., respiration^26^) and environmental contexts (e.g., footfalls on different substrates, weather^27,28^) well compliments the information content of acoustic signals and cues, can provide information on social behavior and context, reproductive status^29^, intraspecific competition and heterospecific interactions. These examples demonstrate the value of taking a comprehensive approach to analyzing acoustic recordings.

When data reflecting the experience of individual animals informs decision-making within the context of conservation interventions, animal-bourne acoustic recording units (ARU) provide an opportunity to collect data regarding heretofore unobservable phenomena. Additionally, by placing the ARU at the focal source, both the emission of sounds and reception of sounds can be assessed in an animal-centric context. This is advantageous in a range of circumstances including social interactions (including parental care), heterospecific interactions, interactions with the environment, and importantly for assessing an animal’s exposure to anthropogenic noise^30^. Received noise levels gained via animal-bourne sensors provide an ideal locale from which to gauge sound exposure levels–the biological context that is most relevant to wildlife disturbance studies^31^. For conservation dependent species, all of these factors can play an important role in assessing and improving management strategies (e.g., restrictions on human use of habitat, and the expression of species typical, and fitness enhancing behaviors of managed individual animals).

Animal-bourne ARU can be used for targeted behavioral monitoring or can be used to establish activity budgets on a more comprehensive scale. For example, for vocalizations that have a recognizable acoustic structure and contextual meaning, pattern recognition methods can be used to extract these events from longer recordings^32^. ‘Smart sensor’ development (i.e., on-board processing and connectivity) could greatly increase the feasibility of acoustic monitoring and reduce the size of data files for transmission^33^. A similar approach can be used for patterned incidental sounds reflecting important behaviors such as feeding or vigilance^25^. For these, recognition of behaviors may be achieved relative to other recorded sounds, as may be the case for resting behaviors, where the relatively extended absence of the sounds associated with movement or feeding may be reasonably assumed to be a signal of resting. Thus, theoretically, activity budgets can be established if preliminary work is done to compile a comprehensive library of behavioral states and their associated acoustic signatures (e.g.^34^).

The giant panda (*Ailuropoda melanoleuca*) is an elusive species of bear that is *vulnerable* to extinction^35^. While this species is dependent upon conservation management for its survival in the wild, available habitat appears to be increasing^36^, and translocation of captive born individuals to the wild is a growing component of the species’ overall conservation strategy^37^. Between 2003 and 2018, the China Conservation and Research Centre for the Giant Panda (CCRCGP) released nine captive born pandas into wild. Protocols for the translocation program include the birth of release candidates in large naturalistic enclosures, and maternal-rearing with little to no human contact. At approximately two years of age, selected candidates are instrumented with GPS tracking collars and released into reserves^37^. While there have been a small number of captive-born giant pandas released to-date, the program has been relatively successful with two documented mortalities of nine total animals released (*H. Zhang, unpublished data*). However, there is currently no evidence that captive-born released individuals have been recruited into the wild breeding population. Additionally, the underlying cause of each post-release mortality has not been identified with certainty, save for one male panda that was presumed to have died from injuries received during intrasexual competition in the breeding season. These scenarios support the notion that pre-release training should be targeted in such a way as to improve the likelihood of survival and recruitment, and that efforts should be made to improve our capacity to monitor the behavior of pandas post-release. Thus, developing post-release monitoring strategies that track both movements and behavior are a priority, especially given the species social and reproductive system, and their exclusive reliance on bamboo for sustenance^38^.

Giant pandas are ideal subjects for animal-bourne acoustic monitoring. A large literature has documented the acoustic ecology of the species in relatively fine detail providing ample contextual information for the range of distinctive vocalizations that pandas emit^29,39–41^. For example, during the breeding season giant pandas females emit vocalizations that convey temporally explicit information to males regarding the timing of impending ovulation^29^ and breeding interest^42^. Males emit vocalizations reflecting copulatory success^43^. During the post-partum period, giant panda cubs emit a range of vocalizations that convey information to panda mothers regarding basic needs or satisfaction^44^. Beyond distinctive acoustic signals, the giant pandas’ near exclusive reliance on bamboo for sustenance means that foraging and food consumption have a relatively consistent acoustic signature. Understanding the degree to which released pandas are encountering human infrastructure and activities is also a priority for understanding the experience of release candidates and the challenges they face^37^. Indeed exposure to noisy anthropogenic activities (e.g., vehicle traffic, resource extraction) may be readily identified and panda specific audibility interpreted^45^ via acoustic monitoring, as could be the sounds of herding activities. While reserves offer a degree of protection and appropriate habitat, human activities are common and pandas may move outside the boundaries of reserves post-release. The most recent National Survey for giant panda documented that, in Sichuan Province, approximately 22% of all sign indicating the presence of giant pandas has been found outside reserve boundaries^46^.

Here we investigated the efficacy of using collar mounted ARUs to monitor giant panda behavior remotely, and we compared activity budgets derived acoustically with those previously generated from wild giant pandas via accelerometers^47^ and through direct visual observation^38^. In the context of the giant panda translocation program, our work has implications for both post-release monitoring and for pre-release training, as understanding the behavior of maternal-mentors is essential to determining the appropriateness of the training context. To maximize the efficiency and repeatability of sound classification, automated identification of sounds based on spectrographic characteristics and amplitude contours is preferred^48^. However, an important starting place for the development of this approach is validating the concept using human listeners for classification then refining identification based on acoustic parameters that can ultimately be classified in an automated application. This work thus represents an important step in developing a reliable and informative approach to monitoring the behavior of giant pandas remotely.

## Results

### Efficacy of acoustic recordings relative to visual observation

We found a high degree of accuracy for decoding acoustic-derived behaviors relative to the video-derived key (Table S1). Overall, most observer-associated Kappa coefficients were greater than 0.91, with a single observer coefficient of 0.81. Further we calculated Kappa coefficients for all 10 behaviors to be above 0.90, indicating a high congruency between the audio sampling method and visual sampling method for each individual behavior (Table S2).

**Figure 1:**
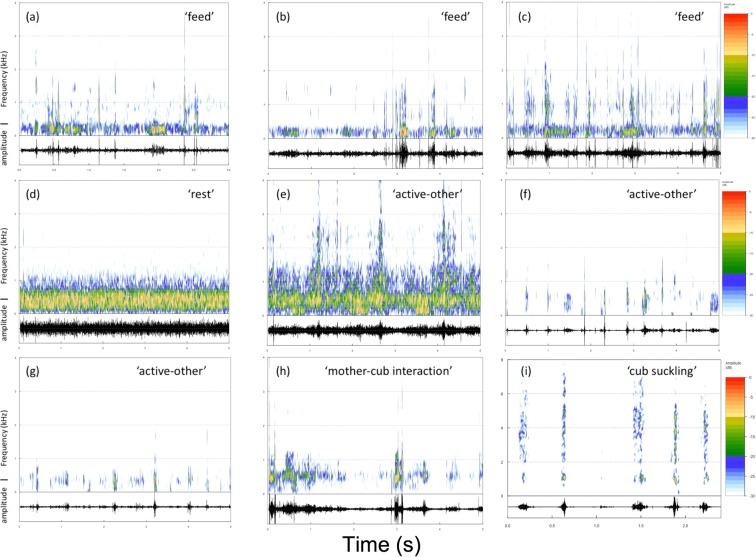
Spectrograms and associated oscillograms of a subset of relevant behaviors that were identifiable by human listeners: (**a**) Feed-bamboo; (**b**) Feed-bamboo shoots; (**c**) Feed-steamed bread; (**d**) Rest; (**e**) Locomotion; (**f**) Stereotypic pace; (**g**) Drink water; (**h**) Mother-cub interaction; (**i**) Cub suckling. Behavioral categories derived via visual inspection of spectrograms and amplitude contours is shown in quotes for each individual behavior.

We further refined our acoustic ethogram by visually inspecting spectrograms for each of the behaviors in our ethogram. For each behavior of interest, we examined 10 randomly selected 5-second recordings for patterns of relative amplitude modulation and spectral energy across frequencies, and found identifiable patterns for 5 behavioral categories: feed (Figure 1a–c), rest (Fig. 1d), ‘active-other’ (Fig. 1e–f), mother-cub interaction (Fig. 1h), and cub-suckling (Fig. 1i). These behaviors and behavioral categories were used in our analyses of activity budgets.

### Activity patterns

**Figure 2:**
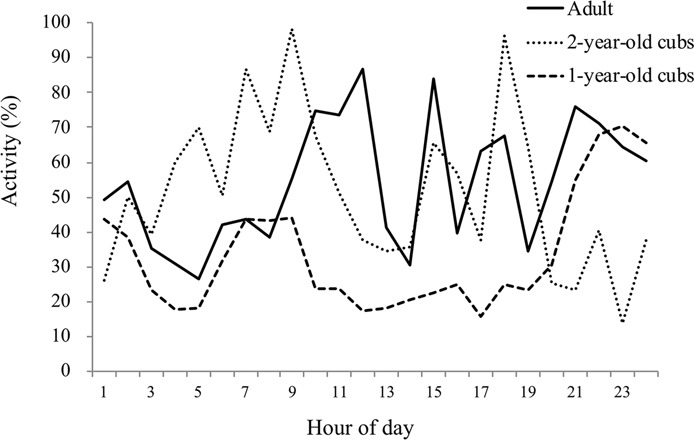
Mean acoustically-derived hourly activity levels for adult, 2-year old and 1-year old giant pandas included in this study. Activity includes all non-rest behaviors.

Daily patterns of activity between the three age classes (adult, 2-year old cub, 1-year old cub) are presented in Fig. 2. Overall, we found no significant difference between individuals in activity level (One-Way ANOVA, F = 1.049, df = 5, P = 0. 43). Adult females had variable periods of activity over 24-hour periods (Kruskal Wallis Test, χ2 = 71.63, df = 23, P < 0. 001). During daytime hours adult females were most active from 1000–1100 h, 1400–1500 h and 1700–1800 h; at night, active periods were between 2000–2100 h and 0100–0200 h. For 2-year old cubs hourly activity levels were similar to adults except for a notably higher level of activity in the early morning hours. Diurnal activity was concentrated around 0600–0900 h and 1500–1800 h, and nocturnal activity peaks are at 0100–0200 h and 0400–0500 h. One-year old cubs were less active than adults or 2-year olds (Kruskal Wallis Test, χ2 = 71.60, df = 23, P < 0. 001), and showed less variation throughout the day, save for one activity peak at 2100–2200 h. There was also a significant degree of individual variation between 1-year old cubs (Kruskal Wallis Test, χ2 = 12.30 df = 4, P = 0.015).

**Figure 3:**
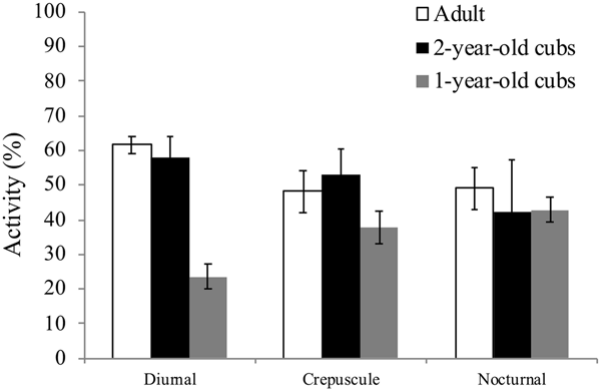
Mean (+/− SEM) acoustically-derived activity levels during diurnal, crepuscular and nocturnal time periods for adult, 2-year old and 1-year old giant pandas.

We analyzed grosser temporal activity patterns by binning the hours of the day into ecologically relevant categories: Diurnal (0800–1800), Crepuscule (0500–0800 and 1800–2100) and Nocturnal (2100–0500) (Fig. 3). Pairwise comparisons showed that the diurnal activity levels of adult females were significantly higher than that of the crepuscule period (t = 2.69, df = 17, P = 0.016). However, we found no significant difference between diurnal and nocturnal, and crepuscular and nocturnal periods. One-year old cubs exhibited an opposite pattern: their activity level was lowest during the day, and this period was significantly different from the crepuscular period (t = −2.93, df = 14, P = 0.011) and nocturnal periods (t = −4.44, df = 14, P = 0.001). One-year old cubs were most active at night, however there was no significant difference between nocturnal and crepuscular periods. Two-year old cubs had a similar pattern as adults, but there were no significant difference among the three time periods suggesting that activity patterns at this age are transitioning from nocturnal to diurnal ones.

**Figure 4:**
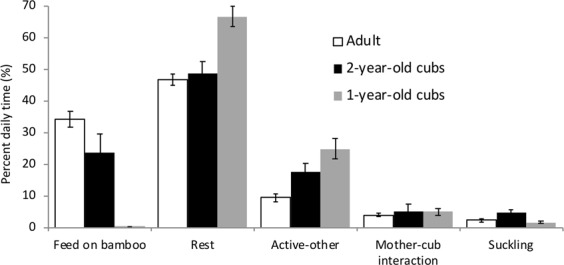
Daily mean (+/− SEM) percent time engaged in behavioral categories for adult, 2-year old and 1-year old giant pandas.

**Figure 5:**
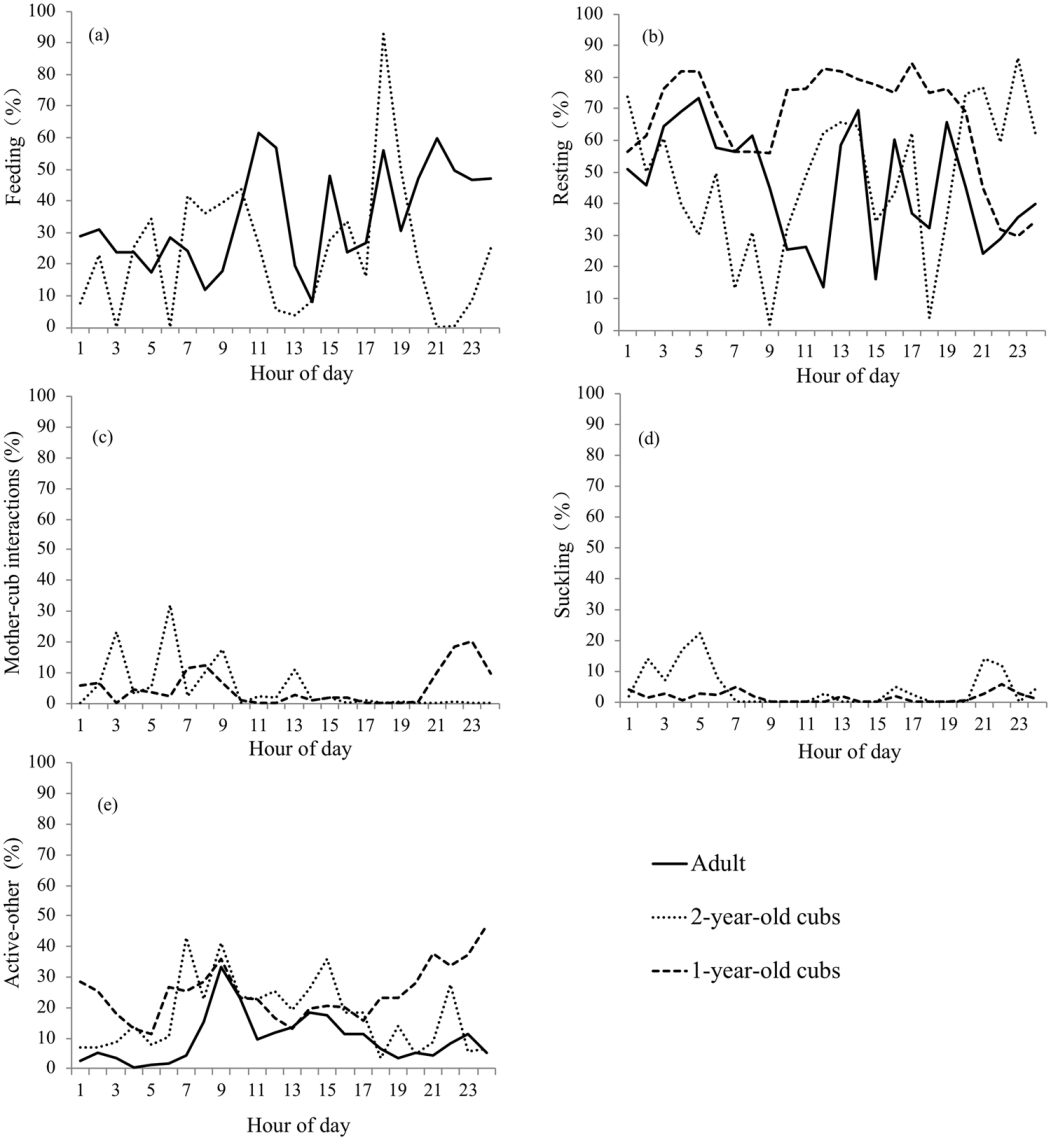
Mean hourly percent time giant pandas spent engaged in each behavioral category as determined from acoustic data. Means represent adult, 2-year old and 1-year old giant pandas respectively and include the following behavioral categories: (**a**) feeding, (**b**) resting, (**c**) mother-cub interactions, (**d**) suckling, (**e**) active-other.

A more finely resolved examination of behaviors showed that feeding, resting, mother-cub interaction, suckling and ‘active-other’ constituted more than 90% of the pandas’ daily time (Fig. 4). Adult resting behavior was not significantly different from that of 2-year old cubs (Student-Newman-Keuls Test q = −0.449, P = 0.73). One-year old cubs spent more time resting than both adults and 2-year old cubs (One-Way ANOVA, F = 17.26, df = 2, P < 0.001), and significantly more than time spent engaged in ‘active-other’ behaviors (Kruskal Wallis Test, χ2 = 48.71, df = 4, P < 0.001). Suckling behavior accounted for a relatively small amount of both 1-year old and 2-year old cubs time budget, however it is likely that this reflects the challenges of capturing this behavior consistently through acoustic observation. Mean hourly patterns of behavior are shown in Fig. 5. Hourly patterns of adult feeding behavior showed less fluctuation than did those of 2-year old cubs. For adult females ‘active-other’ was highest in the morning, peaking at 0900 h. Two-year old cubs showed ‘active-other’ peaks throughout the day, and 1-year old cubs were most active late at night. Mother-cub interactions for 2-year old cubs peaked in the morning hours, while interactions with 1-year old cubs peaked between 2100–2300 h. Again, suckling behavior is seen at relatively low levels, however a clear peak in suckling is seen for 2-year old cubs in the morning hours, between 0200–0700.

## Discussion

We tested the efficacy of using animal-bourne ARU to collect behavioral data on giant pandas, a species *vulnerable* to extinction and for which translocation efforts are at the center of recovery efforts^49^. Our study was focused on mother-cub pairs during the two-year pre-release mentoring period. We paired acoustic recordings with simultaneously collected video recordings in order to validate the accuracy of acoustic identification of behaviors and to test whether the method was repeatable across observers. We found that acoustic recorders provided information on social interactions, feeding behavior (including suckling), and other activities that are important for individual health and survival.

Trained human listeners were able to reliably identify 10 behaviors from acoustic recordings. These behaviors ranged from those that would be expressed in the wild (e.g., feeding on bamboo, locomotion, rest, mother-cub interactions, and cub suckling) to those that are generally unique to the captive setting (e.g., stereotypic pacing, feeding on bread/other). These 10 behaviors provided a high degree of detail regarding the daily activity budgets of the pandas in our study, however, our acoustic analyses graphically demonstrated that grosser behavioral categorization would likely provide more accurate assessment of activity for wild giant pandas, and this could be essential for the practical application of this method in the future, especially if automated acoustical classification were employed. To this end, we reduced our behavioral classification scheme to five categories: feed, rest, mother-cub interaction, suckling, and all other active behaviors (‘active-other’). This degree of resolution provided us with a reliable and appropriately refined behavioral repertoire from which to compare our data with that captured from wild giant pandas^38,47^, and from which to extrapolate the utility of our approach to post-release monitoring.

The acoustic-based daily activity budgets that we derived for adult females were generally consistent with that documented by^47^. However we were able to provide important refinements to the types of behaviors that pandas were engaged in while active, as well as in capturing evidence of cubs and yearlings suckling (albeit a likely underestimation), a behavior that is a critically important for cub survival. Comparing our behavioral data with those documented via direct observation demonstrated similar variation in daily periods of activity^38^, and some degree of individual variation. These comparisons provide us with both validation of the acoustic method of data collection and suggest that the captive reared mentor-adults in our study were engaged in appropriate behavior with dependent young that was consistent with that documented for wild adult pandas.

Appropriate foraging behavior is essential for the survival of translocated individuals^50^, and the giant panda is no exception. From studies of wild giant pandas, foraging time accounts for upwards of 14 hours per day^38^, and the effective processing and feeding on bamboo follows an array of specialized behaviors^51^. This high proportion of time and elaborate processing behavior likely reflect adaptations needed to maximize the energetic uptake from this low calorie food source^52^. However, this sole reliance on bamboo for nutrition is advantageous for acoustic monitoring, reducing the number acoustic signatures associated with energy intake. Our results on time spent feeding on bamboo were comparable with those estimated in previous work^38,47^, and demonstrated the transition of food acquisition from mother’s milk to bamboo, as 2-year old cubs spent an intermediate amount of time feeding on bamboo relative to 1-year old cubs and adults.

Human listeners were able to discriminate the consumption of bamboo shoots from the consumption of bamboo, however, our spectrographic analysis did not reliably distinguish these behaviors, however, future recordings with higher quality ARU may be able to do so. While being able to identify feeding behavior in the general sense from other activities is an important step in monitoring panda behavior, being able to identify bamboo shoot consumption could be an important aspect of post-release monitoring in the future. Bamboo shoots represent an important seasonal food source for giant pandas, offering a different, and ephemerally available, suite of nutrients and calories than the culm and leaves of mature bamboo. As such, for captive-born released giant pandas to thrive in the wild, they must be able to take advantage of this food source when it is available in the early spring^53^.

Relative to the other behaviors in our acoustic ethogram, we were also able to identify resting behavior. This lack of activity, like foraging, is an essential aspect of appropriate giant panda behavior and adequate resting can also reflect a lack of stress^54^. Because of the energetic ‘tightrope’^52^ that giant pandas navigate, adequate rest is essential for maintaining an appropriate energy balance, and may be especially important during the period of maternal care, when energetic demands are intensified both physiologically and behaviorally.

Assessing the degree of maternal care (as expressed here by ‘mother-cub interaction’ and ‘cub suckling’) is important for determining whether cubs are receiving adequate mentorship and nutrition during development. Here we established temporal patterns of maternal care across age-classes and demonstrated that mothers invested more heavily in interacting with 1-year old cubs than with 2-year old cubs, reflecting a redirection of maternal activity away from cubs as they get older and a growing independence that is important for 2-year old cubs as they reach their release/weaning age. The decrease in mother-cub interaction shown by the acoustic data we collected is consistent with a species typical pattern of increasing cub-independence over time^55^.

Conspecific interactions can be key to the success or failure of translocations^37^. Social interactions may result in injury or death if an individual is behaviorally incompetent or non-competitive. Conversely, social interactions are essential for successful recruitment into a population. For species that have distinctive vocal behavior reflecting appropriate courtship and breeding behavior, this can be documented acoustically. For the giant panda this is especially apt. Our data were collected in the post-denning maternal care context. Thus, the signature vocalizations of courtship and breeding were not captured. However, given the extensive analyses of these vocalizations^41,56–59^, it is likely that they would be captured by collar-mounted ARU and identifiable to both human listeners and via acoustic processing and pattern recognition. In the context of mother-cub interactions, understanding the quality of maternal care that is correlated with survivorship of sub-adults post-release will be essential for choosing appropriate mentors and release candidates in the future.

We acknowledge that the configuration of the ARU we used would not be appropriate for long-term deployment on free-ranging giant pandas. However, our findings suggest that the data generated by ARU are appropriate for monitoring giant panda behavior over time in the contexts of post-release monitoring and for pre-release training of pandas whose cubs are destined for translocation. Because of the life-history stage context of the current study (e.g., post-denning maternal care) we were focused on identifying the acoustic signatures of behaviors, not distinctive calls. We also note that the challenges of automated recognition of the complex acoustic signatures of behavior is not trivial, and hope that future efforts will focus intensively on complex signal processing, potentially using ‘deep learning’ or other modes of machine learning. Currently, human listeners are the best tool available for identifying giant panda behaviors, but for longer term deployments wherein larger volumes of data would be generated, this would not be practical, and classification algorithms must be developed using methods of extended machine or deep learning^3^. If human listeners continue to be the most viable option in the near term, the efficacy of this approach may be improved using some form of crowd sourcing^60^, or by targeting specific behaviors of interest, such as courtship vocalizations^61^ or foraging.

Coupling behavioral data at the level of refinement we demonstrate here with high-quality movement data has application during pre-release training, post-release and indeed can greatly improve our understanding of the needs of free-ranging giant pandas. Being able to predict these needs will be key to the success of the translocation program and indeed our ability to predict future population trends in the face of an ever-changing landscape. Over the past 20 years, the number of giant panda reserves has greatly increased^36^ but human activities continue to encroach on panda habitat, and the nature of these activities continues to change, meaning new challenges for the species and a different suite of behavioral impacts and interactions^46^. Additionally, climate change projections suggest that the resources within reserves may not be stable^62,63^, and we may expect to see a lag between panda occupancy and adequate foraging in habitats that are reducing in quality over time. Simply being able to monitor movement along with foraging and active behaviors will greatly improve our understanding of the proximate impact of these threats on species.

Behavioral inadequacy is often identified as a fundamental driver of post-release mortality, lack of recruitment or other scenarios that are indicative of translocation ‘failure’^64^. Thus it is essential that the behavior of individuals is monitored post-release, and that the behavior of mentor conspecifics is consistent with their free-ranging counterparts. These challenges to translocation success make developing methods to monitor the behavior of individual remotely a priority, both in pre-release and post-release contexts. Given the recent advances of bio-logging technologies^10^, remote data transmission^11^ and pattern recognition analyses^65^, we feel that the time has never been better to work towards targeted development of animal-bourne behavioral monitoring techniques.

## Methods

### Study site and subjects

**Figure 6:**
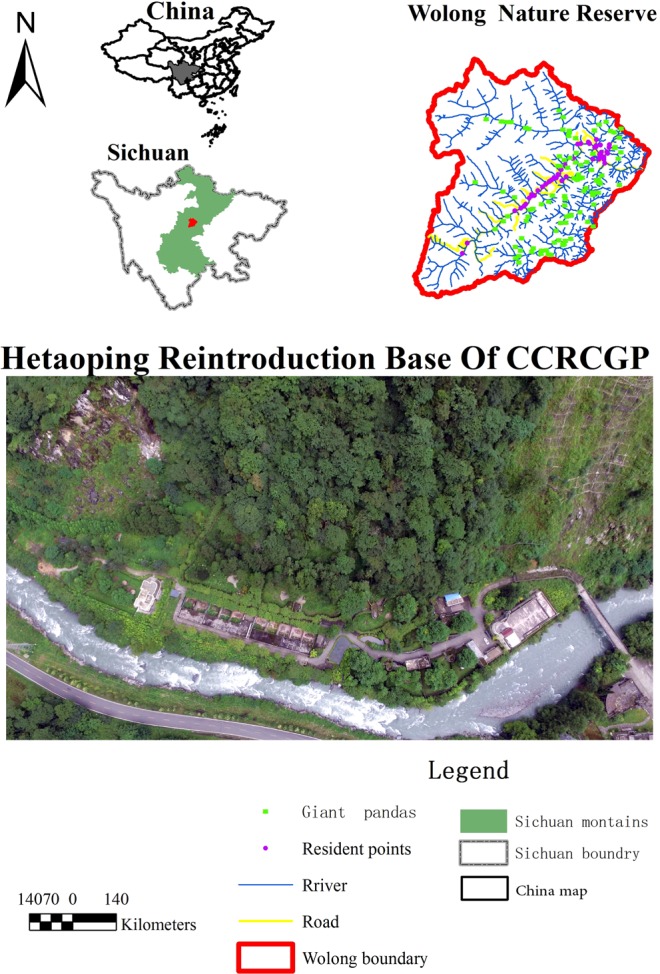
The location of the pre-release training site at Hetaoping, Wolong Nature Reserve, Sichuan, PRC. Giant panda trace sites and residential data are from the 3rd National Survey Report on Giant panda in China. The aerial photo is attributed to the CCRCGP.

This work was carried out at the Hetaoping Base of the China Conservation and Research Center for the Giant Panda (CCRCGP) [102^◦^52′–103^◦^24′E, 30^◦^45′–31^◦^25′N]. Hetaoping Base is located at an elevation of 1820 m.a.s.l. and is situated inside the Wolong Nature Reserve (Fig. 6). As of 2015, the State Forestry Administration in Sichuan estimated that the Wolong Nature Reserve was home to 150 free-ranging giant pandas. The pandas monitored in our study were semi-free ranging, residing in 6 large pre-release acclimation enclosures, measuring 2,000 m^2^ each. These enclosures are used for gestation, birth, rearing and pre-release training of cubs intended for translocation to the wild, and contain topographic features and vegetation typical of the surrounding mountain landscape. Before data collection began, we tested prototypes of the collar mounted ARU on 6 pandas to ensure the functionality of the unit and to ensure that it would be tolerated by pandas. Study subjects for the behavioral validation study included 6 adult females, each of which had a single dependent cub (Table S3). Each mother-cub pair was housed in an individual pre-release acclimation enclosure. Only adult females were fitted with collars, however when cubs were in proximity to their mothers ARU were able to capture cub vocalizations and acoustic signatures of cub behavior (e.g., suckling). All experimental protocols and research activities adhered to regulations of the CCRCGP and adhered to guidelines set forth in the Chinese Regulations and Standards for Captive Animals, and were approved by the CCRCGP.

### Acoustic recording unit (ARU)

**Figure 7:**
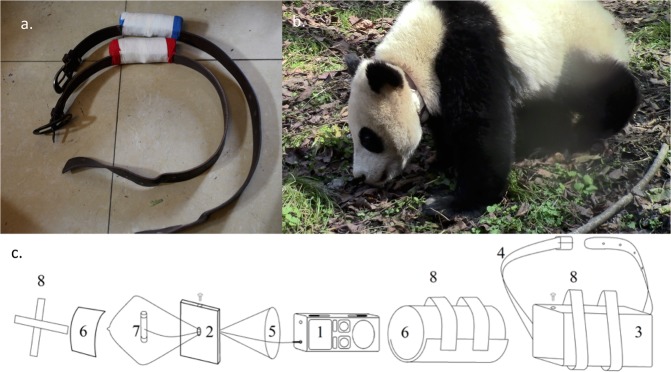
Configuration of collar mounted acoustic recording unit (ARU), (**a**) after being fit on an adult female giant panda (**b**) and schematic drawing the ARU and all its components (*1*. audio recorder; *2*. aluminum cap; *3*. aluminum box; *4*. leather belting; *5*. condom; *6*. gauze; *7*. external microphone; *8*. duct tape strips).

Our study objective was to validate the degree to which acoustic recordings could be used to infer giant panda behavior and habitat use remotely (i.e., without direct visual observations). To this end, we designed a prototype, collar-mounted ARU that could collect good-quality recordings from giant pandas under semi-free ranging circumstances. We configured our ARU using a consumer-grade voice recorder (Sony ICD-PX33M professional digital voice recorder). This model was selected because of its low cost, relatively compact size (38 mm × 114 mm × 21 mm), low energy consumption (3 V, 1500 mA) and light weight (<500 g). The voice recorder was put into a condom to keep moisture out, and then placed into an aluminum case to protect it from impact damage. The space between the condom and the aluminum case was filled with gauze to further stabilize the recorder. A ¼” microphone, with a frequency response of 50 Hz to 20 kHz, was placed outside the aluminum case to maximize recording quality. This microphone was taped securely to the case to minimize movement. The ARU was then attached to an adjustable leather collar using duct tape. The ARU was positioned ventrally, and with the microphone directed toward the panda’s muzzle. Some collars were fitted with an additional layer of fake fur to reduce any collar discomfort and rubbing (Fig. 7). We note that deployment of ARU on free-ranging pandas will require further technological development.

Adult females were trained (via positive reinforcement) to voluntarily allow for the application and removal of the tracking collar. The application and removal procedures took less than 5 minutes to complete each time. To minimize the likelihood that release-candidate cubs were exposed to human presence, application and removal of collars was limited to opportunities when cubs were more than 50 m distant from their mothers and were sleeping. Keepers also wore panda masks and costumes while engaged in collar application and removal. We emphasize that the adult female study subjects were not intended for release to the wild, were comfortable in the presence of humans and were tractable.

Recordings were made using a sampling rate of 44.1 kHz, 192 kbps, and we set digital pitch control, voice operated recording and low-cut filter to ‘off’. Data were saved at 6-hour intervals. All recordings were stored as MP3 file type to maximize storage capacity and battery life. This configuration resulted in a recording capacity of approximately 120 hours. Because of the dense bamboo forests in the giant pandas’ natural habitat, background noise is typically relatively low, thus we selected low noise reduction mode and set the sensitivity of the external microphone to high.

The functionality and configuration of collar-mounted ARU were tested for 14 months, beginning in March 2012. During this time, more than 200 tests were conducted under various conditions, resulting in 824 hours of recordings. From these recordings, we identified 10 behaviors that we felt could be acoustically identified by trained staff, as defined by a well-established giant panda ethogram (Table S4 ^66^). We also developed an acoustically-derived ethogram based on a human-centric description of the sound characteristics of each behavior (Table S4). From these recordings, we trained 10 observers to identify behaviors from acoustic recordings.

### Behavior validation

For behavioral validation of acoustic recordings we paired collar-mounted acoustic recordings with simultaneously collected, time-synchronized video recordings. We used infrared illumination to record video after daylight hours, however 300 hours of video data were excluded from this analysis due to poor-visibility. This data culling resulted in 524 hours of video-synchronized acoustic recordings. We chose to validate acoustic data collected during May because it is a month of high bamboo-shoot availability, relatively high levels of activity and high behavioral diversity^47,67^, thus allowing us to test acoustic identification of the widest range of panda behaviors. Data collection began approximately 24 hours after application of the tracking collar to reduce the impact of the collar fitting process on panda behavior. For the purposes of validation, behavior from video data was recorded using all-occurrence and critical-incident sampling^68^. We quantified accuracy of acoustically-derived behavioral data using an analysis of congruence between the behavior key derived from video recordings and the acoustically-derived data^69^. The order in which recording segments were presented to the observers was randomized. We also assessed the inter-observer reliability between the behavioral observers. Focal animal instantaneous point samples were taken at one-minute intervals for visual and acoustic methods, and we used a Kappa test^69^ to examine the congruency between the classification methods. While we were able to identify the consumption of water acoustically, we did not include drinking in our activity budgets due to the relatively short time frame of its occurrence.

### Acoustic analysis

To facilitate acoustic visualization, we converted MP3 files to WAV format using Audacity 2.1.3 Software^70^. Note that converted MP3 files do not have the same fidelity as native WAV files (but see^71^), therefore no improvement in recording quality was presumed to have occurred through this conversion. We imported converted recording files into Praat v. 5.4.9^72^ to visualize long audio files and to generate shorter exemplar audio clips. To visually illustrate the relative spectral energy present in recordings across frequencies and the temporal patterns of amplitude modulation in recordings, we generated spectrograms and oscillograms using packages SeeWave^73^, warbler^74^ and ggplot2^75^ in R-Studio^76^. We then visually compared the spectral patterns and amplitude contours of each behavior and coalesced individual behaviors scored into broader behavioral categories (Fig. 1): ‘Feed’ reflects all feeding behavior, including feeding on bamboo, bamboo shoots and steamed bread. ‘Rest’ includes non-active behaviors, and can including sleeping or stationary alert behavior. ‘Mother-cub interaction’ includes all behaviors indicative of social interactions between a mother and her dependent cub, including maternal care or social play. ‘Cub-suckling’ only reflects cub nursing behavior, and ‘active-other’ includes all non-rest behavior except for feeding, cub-suckling or mother-cub interaction.

### Data analysis

For all activity budget data, we assessed the normality of data using the Kolmogorov-Smirnov Test^77^ and inspected data for homoscedasticity of variance using Levene’s test^78^. Data that met the assumptions of normality and homoscedasticity of variance were analyzed using one-way ANOVA for difference among group means and we conducted Student-Newman-Keuls Test to examine pair-wise difference^79^. When assumptions of normality were not met, we used the Kruskal-Wallis test^80^. ANOVA was also performed to determine whether there was a significant difference among diurnal, crepuscular and nocturnal activity levels. Significant ANOVAs were followed by paired-samples T-tests to determine significant pair-wise differences. Activity level was defined as the frequency of non-zero occurrence of active behaviors. All non-acoustic data analyses were done using SPSS 19. 0 (Chicago, IL U.S.A).

## Supplementary information

Supplemental materials

## Data Availability

The datasets and materials used in this manuscript are available through request to the corresponding authors.
