# Supplementary material for: Acoustic recordings provide detailed information regarding the behavior of cryptic wildlife to support conservation translocations
Source: Sci Rep. 2019 Mar 26;9:5172. doi: 10.1038/s41598-019-41455-z (PMC6435668; doi:10.1038/s41598-019-41455-z)
Supplement: Supplementary file 1 — Supplemental materials [file 41598_2019_41455_MOESM1_ESM.pdf]

Supplemental Materials:

Acoustic recordings provide detailed information regarding the behavior of cryptic wildlife to support conservation translocations

Xiao Yan[1,3], Hemin Zhang[1], Desheng Li[1], Daifu Wu[1], Shiqiang Zhou[1], Mengmeng Sun[1], Haiping Hu[1], Xiaoqiang Liu[1], Shijie Mou[1], Shengshan He[1], Megan A. Owen[2*], Yan Huang[1*]

[1] China Conservation and Research Centre for the Giant Panda, Wolong, Sichuan, 623006
[2] Institute for Conservation Research, San Diego Zoo Global
[3] Key Laboratory of Biodiversity Science and Ecological Engineering of Ministry of Education, School of Life Sciences, Beijing Normal University, Beijing, China
[*] Corresponding authors

Table S1. Measure of agreement for behavioral scoring, for each of 10 observers

Observer	1	2	3	4	5	6	7	8	9	10
Measure of agreement (Kappa)	0.989	0.952	0.985	0.940	0.981	0.981	0.812	0.912	0.992	0.962
Accuracy (%)	99.3	98.0	99.0	96.8	98.6	98.8	96.4	93.8	99.5	98.5

Table S2. Behavior-specific observer accuracy for acoustically derived data extraction.

Behavior	Kappa	Accuracy (%)	Behavior	Kappa	Accuracy (%)
Rest	0.988	99.4	Feeding: bamboo	0.992	99.3
Active-other	0.994	97.1	Feeding: bamboo shoots	1.00	100.0
Locomotion	0.943	94.8	Feeding: steamed bread	0.976	97.8
Drink water	0.998	100.0	Stereotypic pacing	0.925	87.0
Suckling	0.967	98.0	Mother-cub interaction	0.921	90.5

Table S3. Study subjects included 6 adult females and their 6 dependent young. *denotes wild born female, and best approximation of birthdate.

Name	Gender	Date of birth	Date of experiment	Age at time of experiment (years)
Feifei	♀	08.16.1995	2013.05	18
Yuntao	♂	10.31.2011	2013.05	1.5
Longxin	♀	08.18.2000	2013.05	13
Xinyuan	♀	07.29.2012	2013.05	0.8
Sixue	♀	07.22.2006	2013.05	7
Xuexue	♀	08.15.2012	2013.05	0.7
Caocao*	♀	09.01.2002	2014.05	12
Huajiao	♀	07.06.2013	2014.05	0.8
Yeye	♀	09.25.1999	2014.05	15
Huayan	♀	08.14.2013	2014.05	0.7
Huamei	♀	08.21.1999	2014.05	15
Huarong	♂	07.18.2013	2014.05	0.8

Table S4. Definition and sound characteristics of 11 behaviors of giant panda

Behavior	Ethogram definition*	Acoustic description
Rest	Siting, lying or standing still; awake or asleep.	Ambient noise and/or steady breathing sounds sometimes punctuated with snores.
Locomotion	Quadrupedal movement from point to point.	Rhythmic footfalls often accompanied with breathing sounds that would increase as the pace of the footfalls increased.
Feed: Bamboo	Handling or eating of bamboo, includes processing of bamboo stalk or leaves for consumption.	Biting and chewing sounds punctuated by the sound of bamboo stem breaking apart
Feed: Steamed Bread	Handling or eating steamed bread.	Relatively soft chewing sounds, sometimes accompanied with the sound of swallowing.
Feed: Bamboo Shoots	Handling or eating bamboo shoots.	Biting sounds coupled with the sound of falling bamboo shoot shell and bamboo shoot breaking apart.
Suckling	Suckling on milk secreted by the mother's mammary glands.	Rhythmic sucking sound, punctuated with somewhat regular occurrence of swallowing sound.
Mother-Cub Interaction	Social play between mother and cub, maternal care and grooming.	High frequency of chirp, bleat and moaning vocalizations typical of cub play behavior.
Stereotypic Pace	Highly repetitive locomotion following a pattern that does not serve to move animal in a functional way.	Highly rhythmic footfalls
Drink Water	Consumption of water.	Sucking sound, loud, short duration, and coupled with the sound of running water
Active-Other	Active behavior that does not fall into other defined categories.	Variable non-environmental noises that could not be ascribed to a particular behavior and were indicative of non-rest.
